# Supplementary material for: Haematology dimension reduction, a large scale application to regular care haematology data
Source: BMC Med Inform Decis Mak. 2025 Feb 12;25:75. doi: 10.1186/s12911-025-02899-8 (PMC11823074; doi:10.1186/s12911-025-02899-8)
Supplement: Supplementary file 1 — Supplementary Material 1. [file 12911_2025_2899_MOESM1_ESM.pdf]

Supplementary Material

Table S1 : Preprocessing Clippings.

Measurement	Min	Max
White Blood Cell Count (logscale)	0	6
White Blood Cell Viability Factor	0.75	1.0
Neutrophil Count (logscale)	0	4
% Neutrophils	-	-
Segmented Neutrophil Count (logscale)	0	4
% Segmented Neutrophils	-	-
Lymphocyte Count	0	7
% Lymphocytes	-	-
Lymphocytes Count excluding atypical Lymphocytes	0	7
% Lymphocytes Count excluding atypical Lymphocytes	-	-
Atypical Lymphocyte Concentration	-	-
% Atypical Lymphocyte	-	-
Monocyte Count	0	5
% Monocytes	0	40
Monocyte Count excluding Blasts	0	5
% Monocytes excluding Blasts	0	40
Eosinophil Count	0	2
% Eosinophils	0	20
Basophil Count	0	0.4
% Basophils	0	3
Red Blood Cell Count (optic)	0	8
Red Blood Cell Count (impedance)	0	8
Hemoglobin	0	15
Hemoglobin Concentration (USA-units)	0	25
Mean Corpuscular Volume	40	140
Red Blood Cell Distribution Width	0	25
% Microcytic Red Blood Cells	0	10
% Macrocytic Red Blood Cells	0	25
Mean Corpuscular Hemoglobin Concentration (USA-units)	20	40
Mean Corpuscular Hemoglobin Concentration	0.15	0.3
Mean Corpuscular Hemoglobin (USA-units)	0	50
Mean Corpuscular Hemoglobin	0	3
Hematocrit	0	80
Platelet Count (optic)	0	800
Platelet Count (impedance)	0	800
Mean Platelet Volume	0	20
Plateletcrit	0	1
Platelet Distribution Width	10	25

% Reticulated Platelets	0	20
Reticulocyte Count	0	250
% Reticulocyte Count	0	15
Immature Reticulocyte Fraction	-	-
% Hyperchromic Red Blood Cells	0	2
% Hypochromic Red Blood Cells	0	50
Hemoglobin Distribution Width	0	20
Mean Corpuscular Volume of Reticulocytes	60	160
Mean Corpuscular Hemoglobin of Reticulocytes	0	40
Mean Corpuscular Hemoglobin Concentration of Reticulocytes	-	-
Mean Axial Light Loss of Neutrophils	-	-
%CV Axial Light Loss of Neutrophils	0	6
Mean Intermediate Angle Scatter of Neutrophils	100	175
%CV Intermediate Angle Scatter of Neutrophils	0	8
Mean Polarised Side Scatter of Neutrophils	-	-
%CV Polarised Side Scatter of Neutrophils	0	15
Mean Depolarised Side Scatter of Neutrophils	0	50
%CV Depolarised Side Scatter of Neutrophils	0	30
Mean Fluorescence Signal of Neutrophils	60	100
%CV Fluorescence Signal of Neutrophils	-	-
Mean Axial Light Loss of Lymphocytes	60	140
%CV Axial Light Loss of Lymphocytes	0	12
Mean Intermediate Angle Scatter of Lymphocytes	-	-
%CV Intermediate Angle Scatter of Lymphocytes	0	10
Mean Intermediate Angle Scatter of Red Blood Cells	-	-
%CV Intermediate Angle Scatter of Red Blood Cells	0	3
Mean Fluorescence signal of Red Blood Cells	60	110
%CV Fluorescence signal of Red Blood Cells	0	30
Mean Intermediate Angle Scatter of Platelets	100	190
%CV Intermediate Angle Scatter of Platelets	10	25
Mean Polarised Side Scatter of Platelets	100	150
%CV Polarised Side Scatter of Platelets	0	30
Mean Fluorescence signal of Reticulocytes	100	175
%CV Fluorescence signal of Reticulocytes	0	25

Table S2 : Results of the Cosine-fit for diurnality. The dimension reductions for this result were extracted from a sample of 100.000 measurements, repeated 10 times.

Reduction technique	component	amplitude	offset	bias	p-value
PCA	0	3.26 ± 0.051	0.80 ± 1.44	0.67 ± 1.55	$1e-16$
	1	1.00 ± 0.016	-1.85 ± 0.033	-0.43 ± 0.012	$1e-16$
	2	1.07 ± 0.041	-0.80 ± 0.021	0.20 ± 0.019	$1e-16$
	3	1.57 ± 0.037	-0.26 ± 0.017	0.73 ± 0.018	$1e-16$
	4	0.40 ± 0.009	-1.61 ± 0.040	-0.12 ± 0.009	$1e-16$
	5	0.82 ± 0.011	0.56 ± 0.019	0.52 ± 0.006	$1e-16$
GRP	0	2.01 ± 1.15	-1.08 ± 1.94	0.11 ± 1.31	$1e-16$
	1	1.44 ± 1.23	-0.35 ± 1.52	0.21 ± 0.82	$1e-16$
	2	1.70 ± 0.84	0.27 ± 1.61	0.38 ± 0.96	$1e-16$
	3	1.51 ± 0.78	1.33 ± 1.75	0.0046 ± 0.90	$1e-16$
	4	1.02 ± 0.54	0.81 ± 1.76	-0.058 ± 0.55	$1e-16$
	5	1.44 ± 0.10	1.28 ± 1.88	-0.37 ± 0.73	$1.5e-3$
UMAP	0	0.13 ± 0.096	0.48 ± 2.04	2.24 ± 3.67	$1e-16$
	1	0.15 ± 0.043	-1.14 ± 2.18	1.24 ± 1.50	$1e-16$
	2	0.19 ± 0.070	1.69 ± 1.92	9.31 ± 1.86	$1e-16$
	3	0.87 ± 0.510	-0.95 ± 0.85	5.20 ± 1.45	$1e-16$
	4	0.70 ± 0.509	1.31 ± 1.42	4.82 ± 1.54	$1e-16$
	5	0.11 ± 0.059	-1.20 ± 1.30	1.17 ± 2.05	$1e-13$
TriMap	0	15.82 ± 0.24	0.74 ± 1.44	3.55 ± 8.1	$1e-16$
	1	5.10 ± 0.06	-1.21 ± 0.024	2.26 ± 0.125	$1e-16$
	2	5.05 ± 0.20	-0.61 ± 0.014	2.86 ± 0.133	$1e-16$
	3	7.94 ± 0.19	-0.38 ± 0.010	5.42 ± 0.147	$1e-16$
	4	1.40 ± 0.08	-0.98 ± 0.059	0.52 ± 0.069	$1e-16$
	5	0.73 ± 0.053	0.32 ± 0.060	0.54 ± 0.029	$1e-16$
PaCMAP	0	2.15 ± 0.033	0.71 ± 1.440	0.43 ± 0.99	$1e-16$
	1	0.54 ± 0.018	-1.19 ± 0.040	-0.069 ± 0.015	$1e-16$
	2	0.82 ± 0.024	2.65 ± 0.025	-0.35 ± 0.01	$1e-16$
	3	0.99 ± 0.020	2.85 ± 0.017	-0.50 ± 0.015	$1e-16$
	4	0.38 ± 0.030	2.26 ± 0.075	-0.081 ± 0.020	$1e-16$
	5	0.19 ± 0.041	-1.72 ± 0.090	-0.047 ± 0.016	$1e-16$
Neutrophil fraction	n/a	7.3 ± 0.1	0.1 ± 0.01	68.9 ± 0.08	$1e-16$
Eosinophil fraction	n/a	0.24 ± 0.002	-2.8 ± 0.01	0.85 ± 0.002	$1e-16$

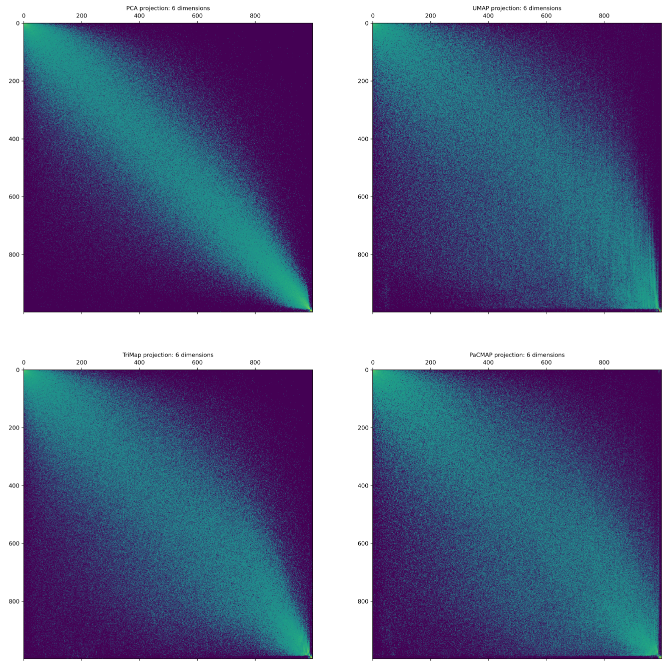

Figure S1 : Comparison of Q-matrix graphs between different dimension reductions of 6 dimensions.

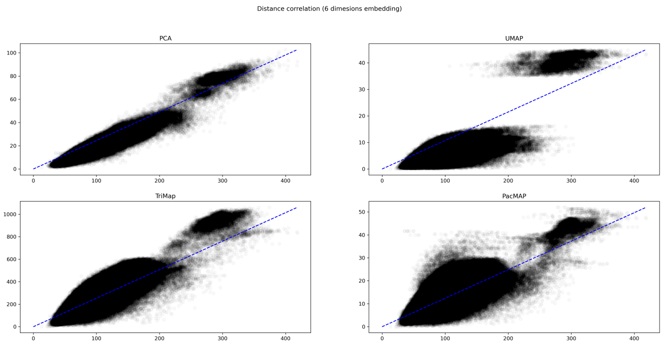

Figure S2 : Correlations of distances between high-dimensional spaces and low-dimensional spaces (6 dimensions) of different dimension reductions

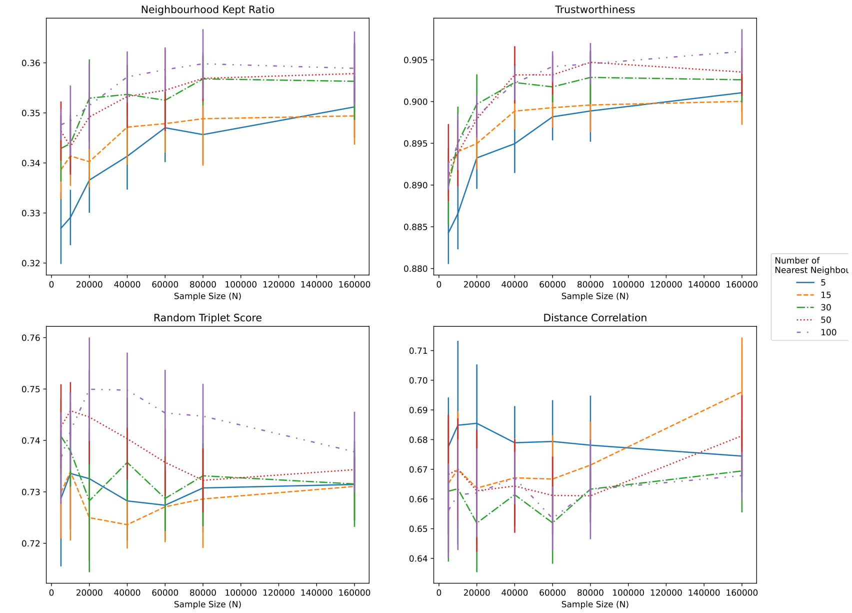

Figure S3 : Evaluating PaCMAP with quality metrics across different numbers of neighbours and numbers of outliers, along with the 95% SE for each sample size and number of neighbours.

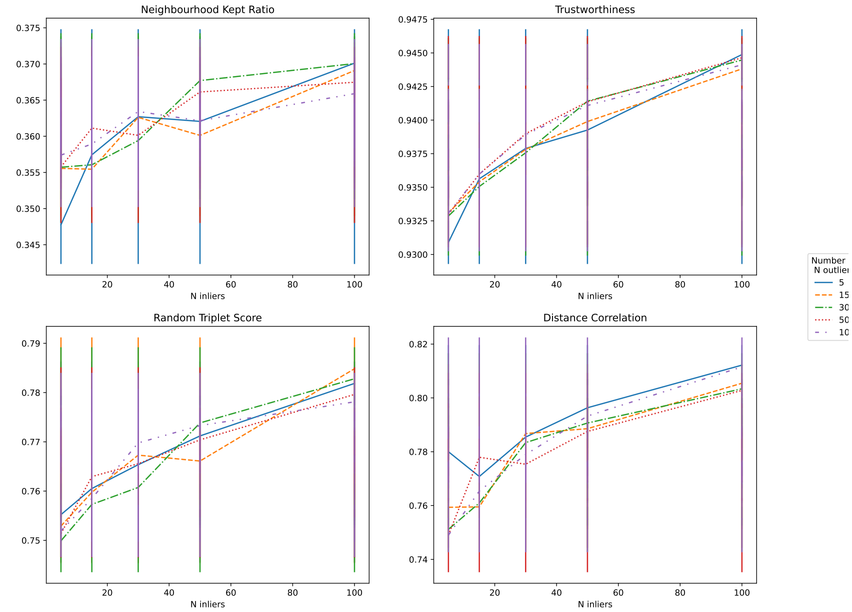

Figure S4 : Evaluating TriMap with quality metrics across different numbers of neighbours and numbers of outliers, along with the 95% for each number of inliers and outliers.

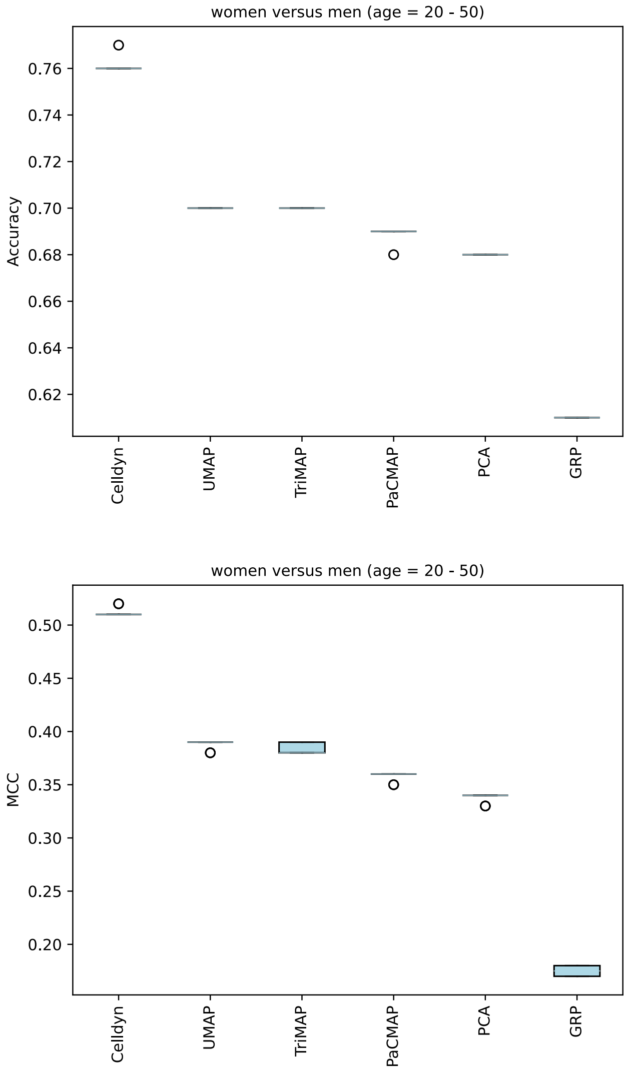

Figure S5 : Predictive performance for sex. n = 170,000 for training; n = 30,000 for validation; n *folds* = 10. 'Celldyn' refers to the original data. Circles represent an outlier in performance

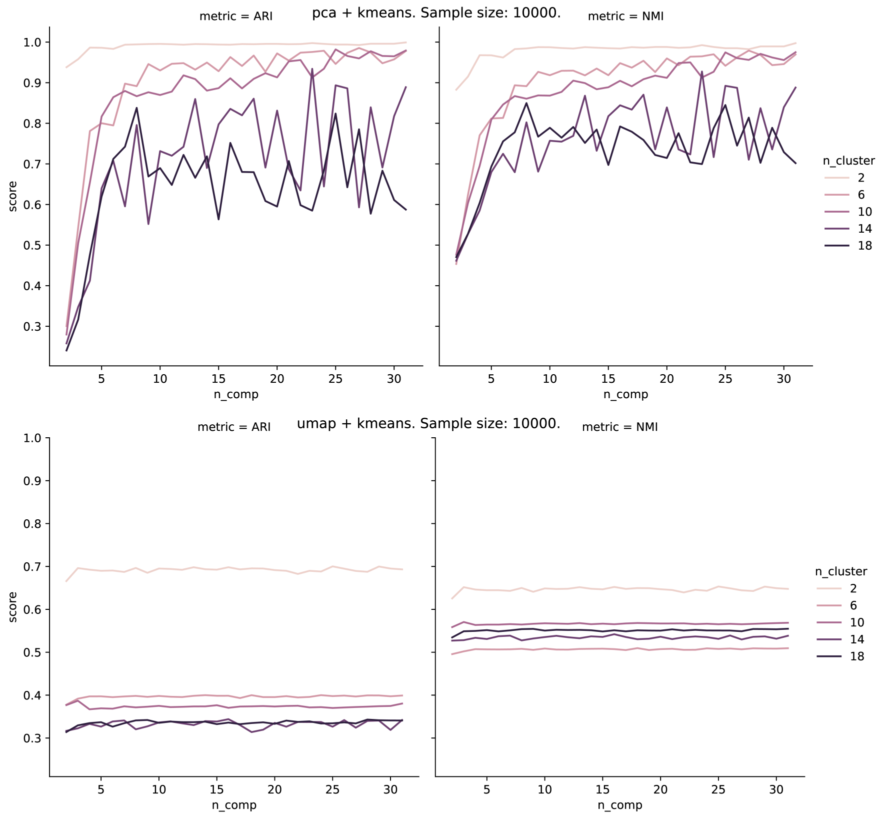

Figure S6 : Kmeans cluster quality of the dimension reductions as expressed by the Normalised Mutual Information (NMI) and Adjusted Rand Index (ARI) using different. Here we drew 10,000 samples for the dimension reductions. Prior to the dimension reduction, z-score scaling was applied to the data.

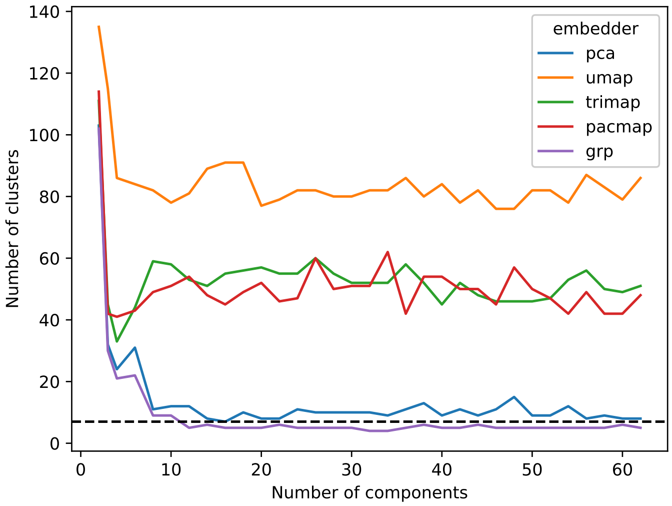

Figure S7 : Number of clusters extracted using HDBSCAN. Here, dimension reduction was performed with PCA, UMAP, TriMap, PaCMAP, or GRP on 10.000 samples.

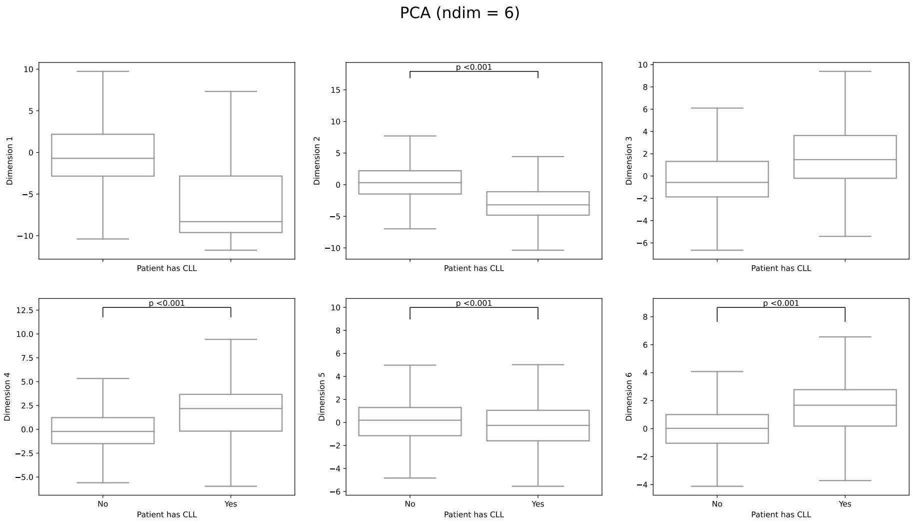

Figure S8 : Chronic lymphocytic leukemia
patient data in the PCA dimension reduction (6 dimensions)

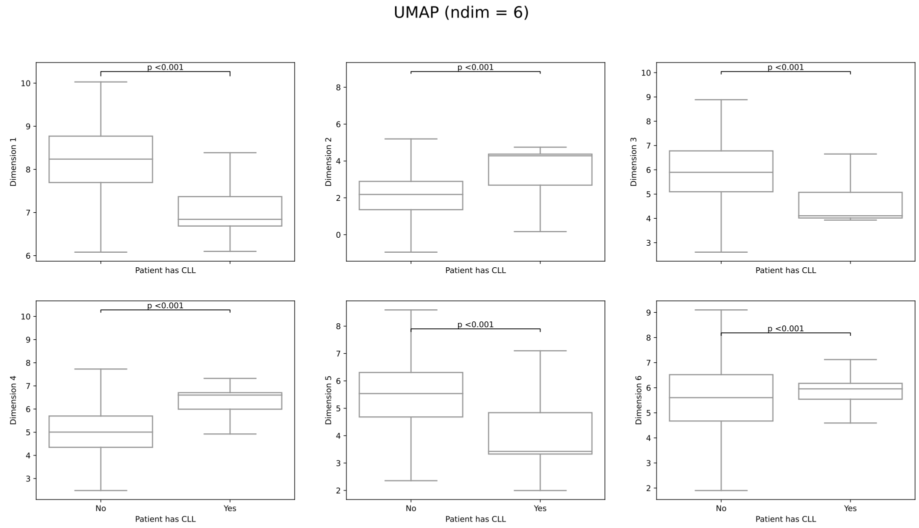

Figure S9 : Chronic lymphocytic leukemia
patient data in the UMAP dimension reduction (6 dimensions)

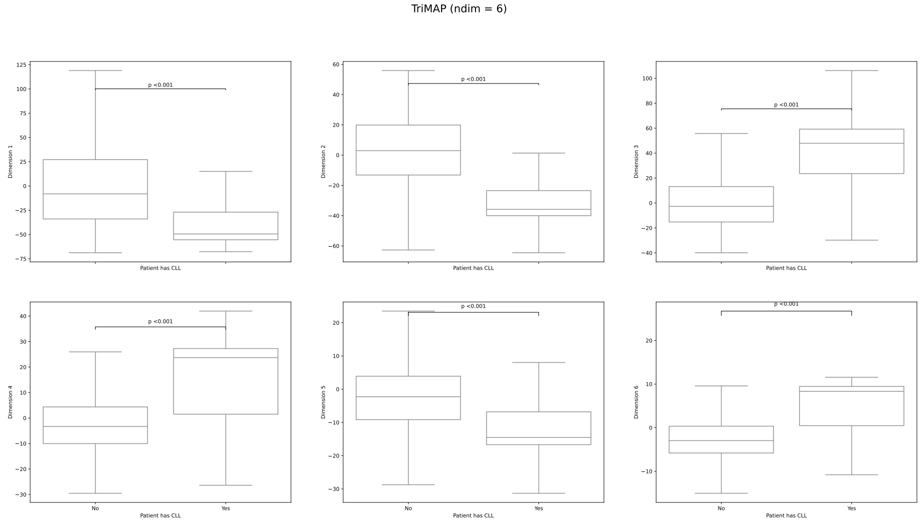

Figure S10 : Chronic lymphocytic leukemia
patient data in the TriMap dimension reduction (6 dimensions)

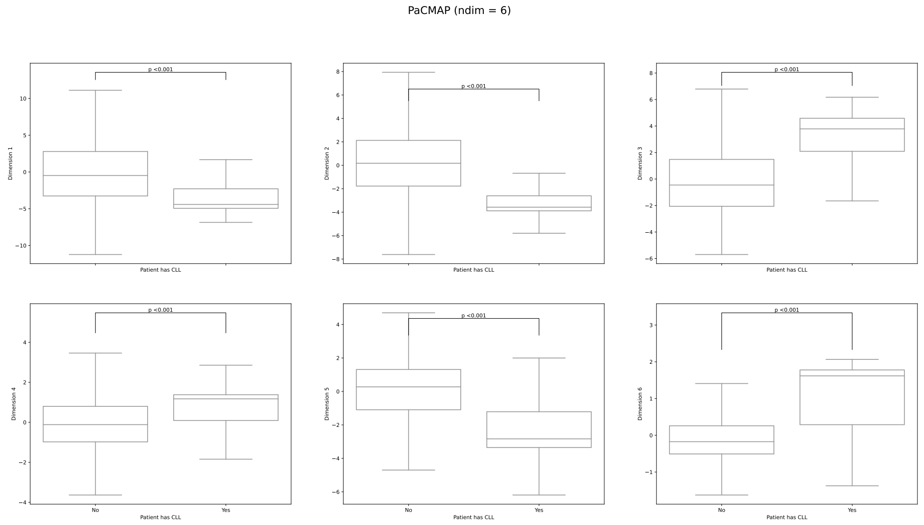

Figure S11 : Chronic lymphocytic leukemia
patient data in the PaCMAP dimension reduction (6 dimensions)

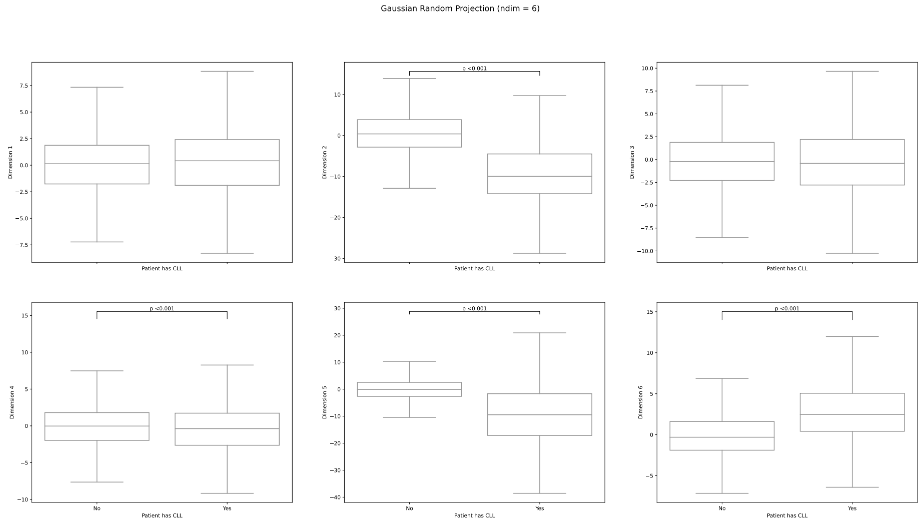

Figure S12 : Chronic lymphocytic leukemia
patient data in the GRP dimension reduction (6 dimensions)

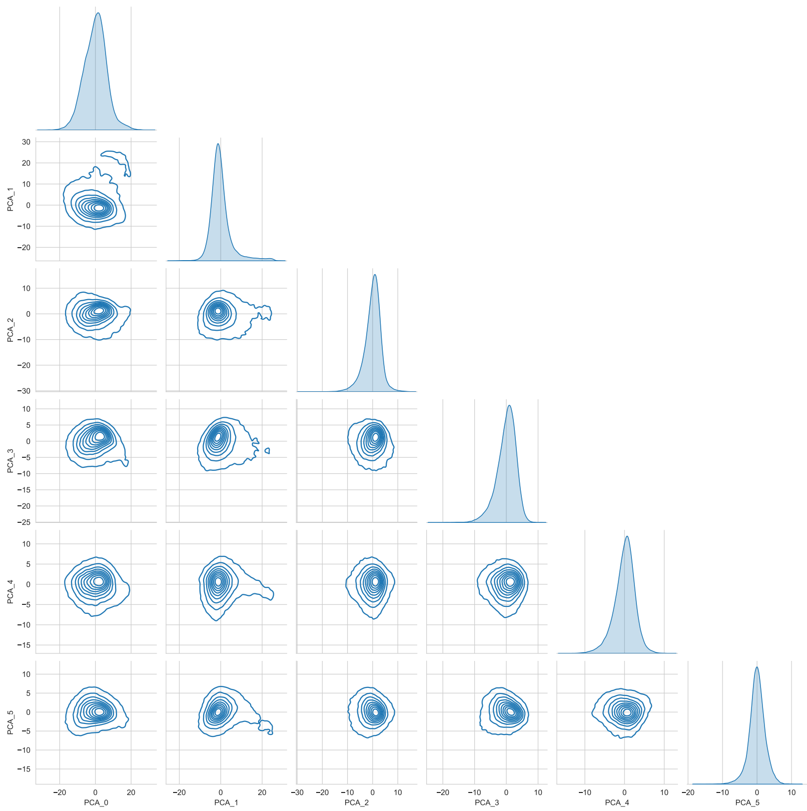

Figure S13 : Density pairplot for the first 5 dimensions after PCA dimension reduction

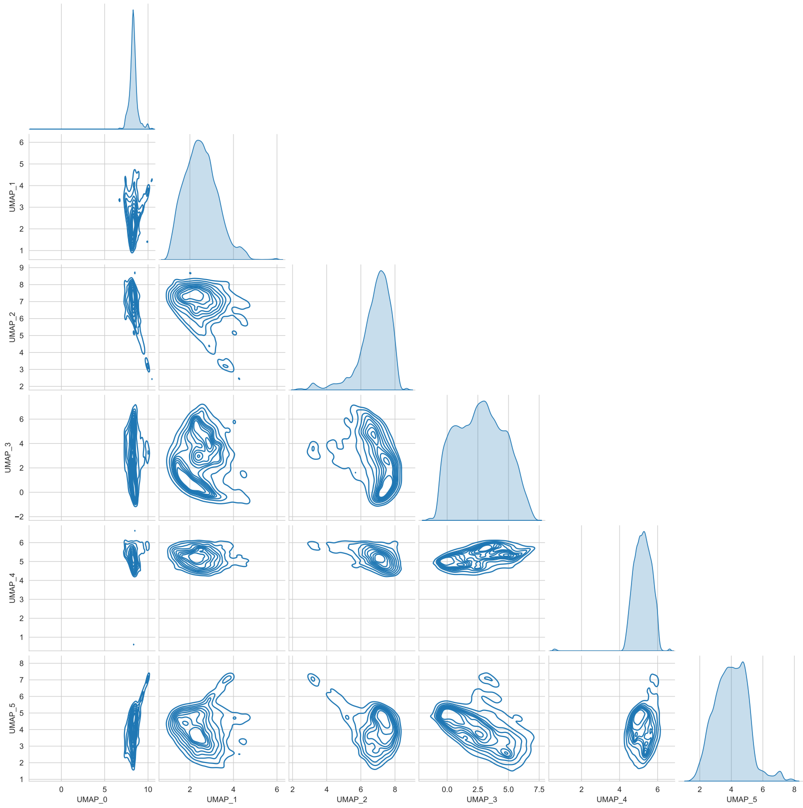

Figure S14 : Density pairplot for the first 5 dimensions after UMAP dimension reduction

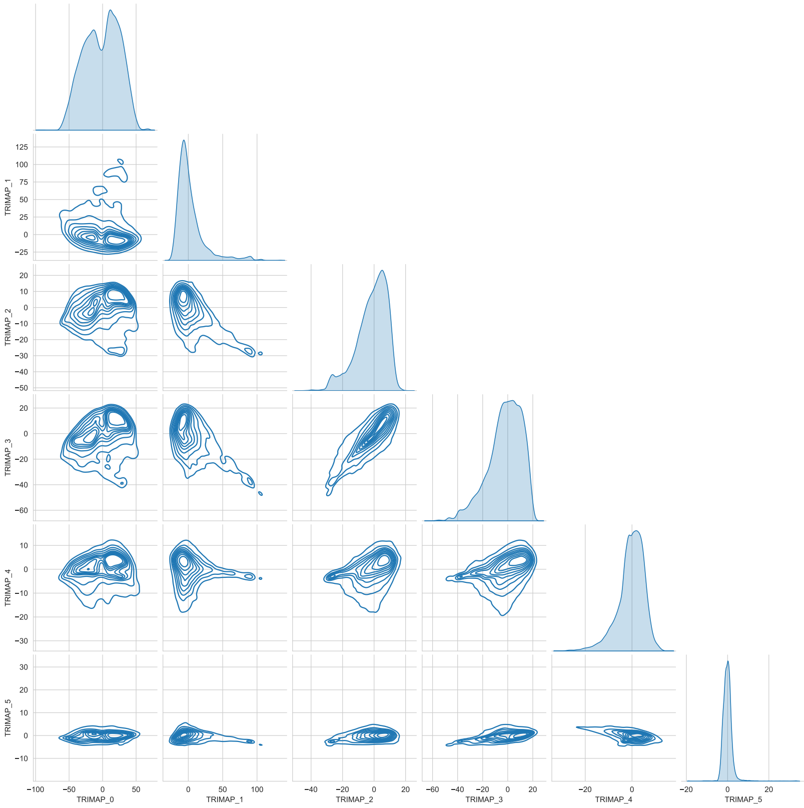

Figure S15 : Density pairplot for the first 5 dimensions after TriMap dimension reduction

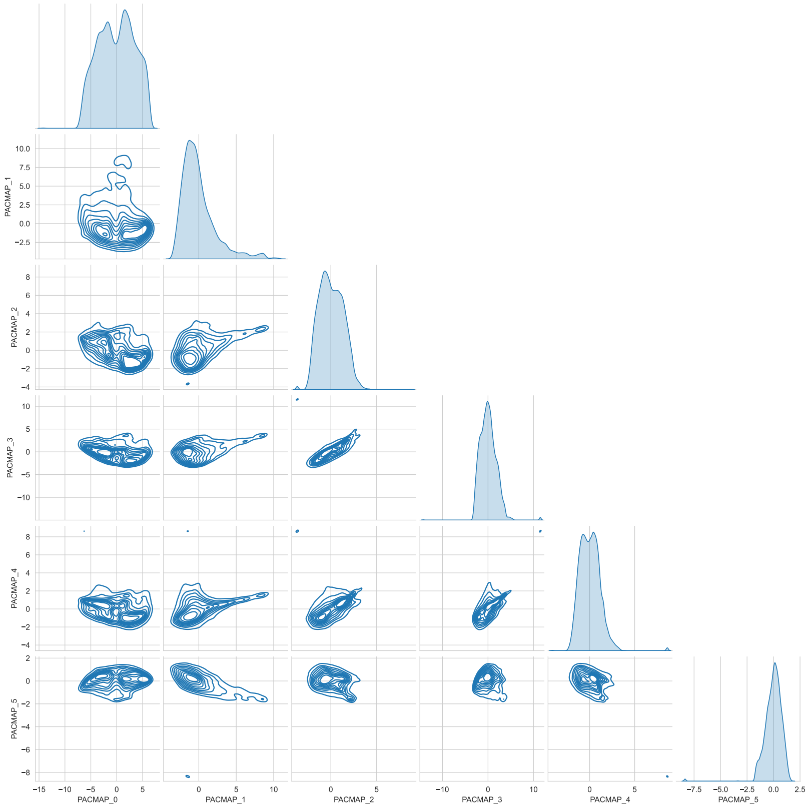

Figure S16 : Density pairplot for the first 5 dimensions after PacMAP dimension reduction

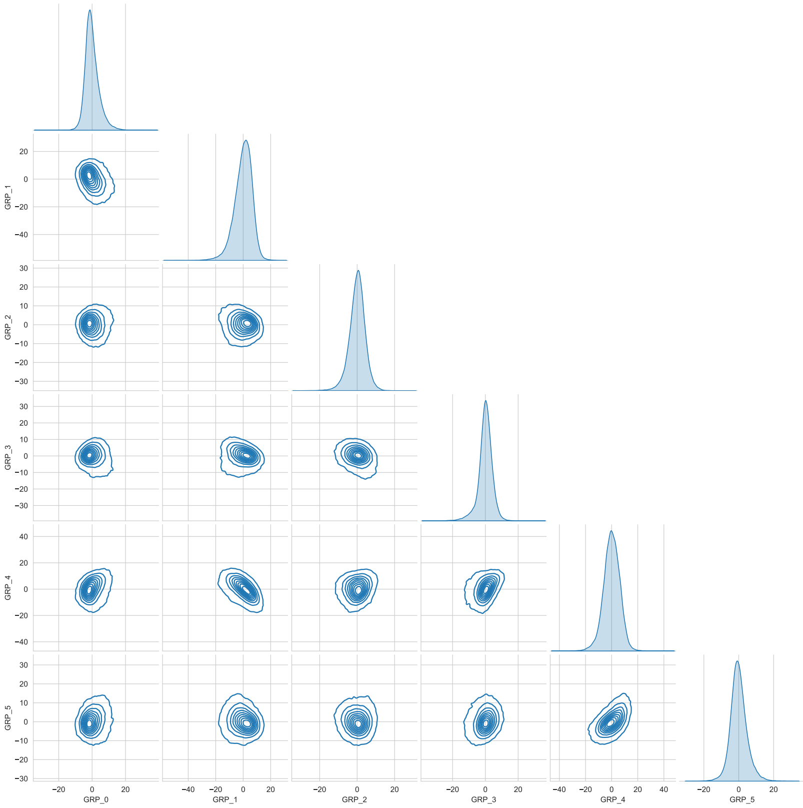

Figure S17 : Density pairplot for the first 5 dimensions after GRP dimension reduction
